# Identification of HMOX1 as a Critical Ferroptosis-Related Gene in Atherosclerosis

**DOI:** 10.3389/fcvm.2022.833642

**Published:** 2022-04-14

**Authors:** Daiqian Wu, Qian Hu, Yuqing Wang, Mengying Jin, Ziqi Tao, Jing Wan

**Affiliations:** ^1^Department of Cardiology, Zhongnan Hospital of Wuhan University, Wuhan, China; ^2^Department of Gastroenterology, Zhongnan Hospital of Wuhan University, Wuhan, China; ^3^School of Basic Medical Sciences, Wuhan University, Wuhan, China

**Keywords:** atherosclerosis, ferroptosis, HMOX1, heme oxygenase-1, vascular smooth muscle cells

## Abstract

Ferroptosis is a novel form of programmed iron-dependent cell death. The ferroptosis-related genes (FRGs) have been recognized as biomarkers for cancers. Increasing evidence has indicated that ferroptosis is involved in the process of atherosclerosis. However, the potential FRGs used for the diagnosis, prognosis and therapy for atherosclerosis are still unclear. We aimed to identify the ferroptosis-related differentially expressed genes (DEGs) of atherosclerosis. We downloaded the mRNA-sequencing data of patients with atherosclerosis from the Gene Expression Omnibus (GEO) database. HMOX1 was identified as an essential ferroptosis-related DEG by bioinformatic analysis of the GSE28829 and GSE43292 datasets. The pro-ferroptotic effect of HMOX1 was validated through cell experiments. Then we conducted a single-gene analysis of HMOX1 and found that high-expression of HMOX1 in atherosclerotic plaques was accompanied by matrix metalloproteinases (MMPs) producing and M0 macrophages infiltration. Taken together, our present study suggested HMOX1 as a potential diagnostic biomarker for atherosclerosis and provided more evidence about the vital role of ferroptosis in atherosclerosis progression.

## Introduction

Atherosclerosis, a complex chronic disease caused by the formation of atherosclerotic plaques, is the underlying pathological process of coronary artery disease (CAD) and cerebrovascular disease (1), and is the major cause of death and disability worldwide (2). Vascular smooth muscle cells (VSMCs) play a pivotal role in atherosclerosis (3, 4). Evidence has revealed that cell death of VSMCs is involved in atherosclerotic plaque progression (5, 6). Multiple stimuli such as oxidized lipids, cytokines, DNA damage, hypoxia and reactive oxygen species (ROS) can drive apoptosis, necrosis and senescence of VSMCs, which lead to plaque progression and vulnerability (7).

Ferroptosis is a novel form of programmed cell death characterized by cellular free iron accumulation and lipid peroxidation (8). The relationship between iron and atherosclerosis has been widely studied in the past 40 years, and iron overload is identified as a crucial risk factor of atherosclerosis (9, 10). Recent evidence revealed that ferroptosis was involved in the progression of atherosclerosis (11, 12). As the earliest studies of ferroptosis focused on cancers, some ferroptosis-related genes (FRGs) have been recognized as biomarkers for the diagnosis, prognosis and therapy for many cancers (13–15). However, the potential role of FRGs in the diagnosis, prognosis and therapy for atherosclerosis was not fully clarified.

In this study, we integrated the atherosclerosis datasets from Gene Expression Omnibus (GEO) and the FRGs obtained from GeneCards to identify reliable ferroptosis-related differentially expression genes (DEGs) in atherosclerosis. Then, we recognized HMOX1 as an important biomarker and verified its pro-ferroptotic effect in human aortic smooth muscle cells (HASMCs) experimentally. Single-gene analysis was conducted to investigate enriched pathways. Protein-protein interaction (PPI) networks were constructed, and co-expression analyses of proteins were performed to find regulatory relationships. Immune cells infiltration degrees were determined through single-sample GSEA (ssGSEA) method. Overall, we have unveiled a potential ferroptosis biomarker for the progression of atherosclerosis.

## Materials and Methods

### Identification of the Key Ferroptosis-Related DEGs

The FRGs were obtained from GeneCards website (http://www.genecards.org/) (16) with the searching keyword “ferroptosis.” An association score was used to indicate the correlation between genes and ferroptosis activity. Larger scores represented stronger associations. Genes with the score > 2 were considered FRGs (All FRGs were listed in Supplementary Table 1).

Two atherosclerosis-related microarray datasets, GSE28829 (17) and GSE43292 (18), were downloaded from the GEO database. Data analysis was performed using R project (R version 4.0.3). Firstly, the probe names in the gene expression profiles were converted into gene names according to platform annotation files. Secondly, we picked out DEGs using “limma” package (adjust *p*-value <0.05 and | log2FoldChange | > 1).

The key ferroptosis-related DEGs were identified using the online Venn diagrams drawing tool (http://bioinformatics.psb.ugent.be/webtools/Venn/).

### Single-Gene Analysis for the Ferroptosis-Related DEG

The advanced atherosclerosis samples in GSE28829 and atherosclerosis samples in GSE43292 were separately divided into two groups according to the expression levels of HMOX1. DEGs of the two groups (HMOX1 high- and low-expression groups) were screened using the same methods and parameters mentioned above. Using “clusterProfiler” R package, we conducted GeneOntology (GO) (19) and Kyoto Encyclopedia of Genes and Genomes (KEGG) (20) analyses of the DEGs. The PPI analysis was performed using the STRING database (STRING v11.0) (https://string-db.org/) (21). Interactions between DEGs with a minimum interaction score > 0.4 were visualized on the networks. Then, the PPI networks were analyzed by Cytoscape 3.5.1. We used CytoHubba plugin to calculate Maximal Clique Centrality (MCC) scores and selected the top 10 hub genes (22). Venn diagrams drawing tool was applied to identify the common hub genes among GSE28829 and GSE43292. The correlation between hub genes was analyzed using logistic regression.

### Immune Infiltration Analysis

Using “GSVA” and “GSEABase” R packages, ssGSEA was performed to obtain scores for 29 immune gene sets in each sample (23). We performed a hierarchical cluster analysis to divide samples into groups with different immune infiltration degrees (24). Expression levels of HMOX1 between high and low immune score groups were analyzed. In addition, “CIBERSORT” package was used to quantify the 22 immune cells in all samples and samples with CIBERSORT *p*-value <0.05 were enrolled in more detailed analysis.

### Cell Culture and Treatment

HASMCs were purchased from China Center for Type Culture Collection (CCTCC, GPC0113). Cells were cultured in Dulbecco Modified Eagle medium (DMEM, Hyclone) supplemented with 10% fetal bovine serum (FBS, Gibco) and 1% penicillin/streptomycin (Hyclone) in an incubator with 5% CO_2_ at 37°C. After reaching a density of 70–80%, cells were treated with erastin (HY-15763, MedChemExpress), zVAD-fmk (HY-16658B, MedChemExpress), necrosulfonamide (HY-100573, MedChemExpress), 3-Methyladenine (3MA, HY-19312, MedChemExpress), Zinc Protoporphyrin (ZnPP, HY-101193, MedChemExpress), hemin (HY-19424, MedChemExpress) and Deferoxamine (DFO, Ba33112, MedChemExpress) for 24 h, respectively. Human monocytic cell line THP-1 was acquired from CCTCC (GDC0100) and cultured in Roswell Park Memorial Institute 1640 medium (RPMI 1640; Hyclone) supplemented with 10% FBS (Gibco) and 1% penicillin/streptomycin (Hyclone) in an incubator with 5% CO_2_ at 37°C.

### Cell Viability Assay

Cells (3000 cells/well) were seeded in 96-well plates and cultured for 24 h to adhere to the wall. After different treatments, CCK-8 solution (10 μL/well) was added and the plate was placed in 37°C incubation for another 2 h. Subsequently, cell viability was evaluated by measuring the optical absorbance at 450 nm using an ELx808TM Absorbance Microplate Reader (BioTek, USA).

### Lipid Peroxidation Assay

Cells (2.0 ×10^5^ cells/well) were seeded in 6-well plates and treated with hemin (85 μM) for 24 h. One μL of BODIPY-C11 (D3861, Invitrogen, USA) stock solution (10 mM) per well was added and wells were mixed by shaking the plates. Then the plates were returned to the incubator and cells were stained with BODIPY-C11 for 30 min. All the media and cells, including adherent and floating cells, were collected into 15 mL tubes. After centrifuging the tubes and removing the supernatants, cell pellets were washed using 2 mL phosphate buffered solution (PBS). Finally, cell pellets were resuspended with 500 μL PBS. The fluorescence of oxidized C11 (FL1 channel) was detected using a cytoFLEX flow cytometer (Beckman Coulter, USA). The ratio of the FITC-positive population was calculated using the FlowJo software (v 10.4).

### Quantitative Reverse Transcription Polymerase Chain Reaction (qRT-PCR)

Total RNA was isolated using HiPure Unviersal RNA Mini Kit (Magen, China) and RNA concentration was measured using a Nanodrop 2000 spectrophotometer (Thermo Fisher Scientific, USA). RNA was reverse transcribed to cDNA using TOYOBO ReverTra Ace Kit (TOYOBO, Japan). The cDNA was used as a template for the qPCR reaction. The qPCR process was performed using the UltraSYBR Mixture (CWbio, China) on CFX96 Real-time fluorescence quantitative PCR instrument (Bio-rad, USA). GADPH was used to normalize the expression of HMOX1. Primers synthesized by TSINGKE Biological Technology (Wuhan, China) were listed in Supplementary Table 2.

### Western Blot Analysis

Total protein was extracted using RIPA buffer (Beyotime, China). The protein concentration was measured by using a BCA protein assay kit (Beyotime, China). Protein was isolated by sodium dodecyl sulfate-polyacrylamide gel electrophoresis (SDS-PAGE). Gels were blotted using a Trans-Blot Turbo blotting system (Bio-Rad, USA) and then transferred onto polyvinylidene fluoride membranes. The membrane was blocked with Protein Free Rapid Block Buffer (PS108, EpiZyme, China) for 10 min and incubated with primary antibodies against heme oxygenase-1 (HO-1, 1:1000, #43966, Cell Signaling Technology, USA), glutathione peroxidase 4 (GPX4, 1:1000, ab125066, Abcam, USA), transferrin receptor (TFRC, 1:1000, #13113, Cell Signaling Technology, USA), matrix metallopeptidase 9 (MMP9, 1:2000, ET1704-69, HuaBio, China) and β-actin (1:1000, #3700, Cell Signaling Technology, USA) at 4°C overnight, followed by incubation of anti-rabbit/mouse Horseradish peroxidase (HRP)-conjugated secondary antibody (1:5000, Promoter, China) at room temperature for 2 h. Protein bands were visualized using a protein imaging system and Ultra-sensitive ECL Chemiluminescence Kit (Beyotime, China). The quantification of Western blot data was performed using Image J software.

### Adhesion Assay

Monocyte adhesion assay was performed as previously described (25). Briefly, HASMCs were cultured in 6-well plates (2.0 ×10^5^ cells/well) until 50–60% confluence and treated with DMSO (as control) or erastin (2 μM) for 24 h before the stimulation by 10 ng/ml TNFα (PeproTech, USA) for 6 h. The THP-1 monocytes were labeled with 5 μM Calcein-AM (Beyotime, China) for 30 min in serum-free RPMI 1640 (Hyclone) according to the manufacturer's instruction. The pre-labeled THP-1 cells (7.5 ×10^5^ cells/well) were incubated with HASMCs for 1 h at 37°C. Non-adherent cells were removed by gently washing with PBS five times. Images of attached cells were acquired using a fluorescence microscope (Olympus, Japan), and adherent cells were counted by Image J software.

### Statistical Analysis

Data were presented as mean values ± standard error of mean (SEM) from at least three independent experiments and analyzed with GraphPad Prism 8.4.3 (San Diego, CA). Statistically significant differences between groups were calculated by student's two-tailed *t*-test and *p* < 0.05 was considered to be significant.

## Results

### HMOX1 Is an Essential Ferroptosis-Related DEG Identified From Atherosclerosis Datasets

**Figure 1:**
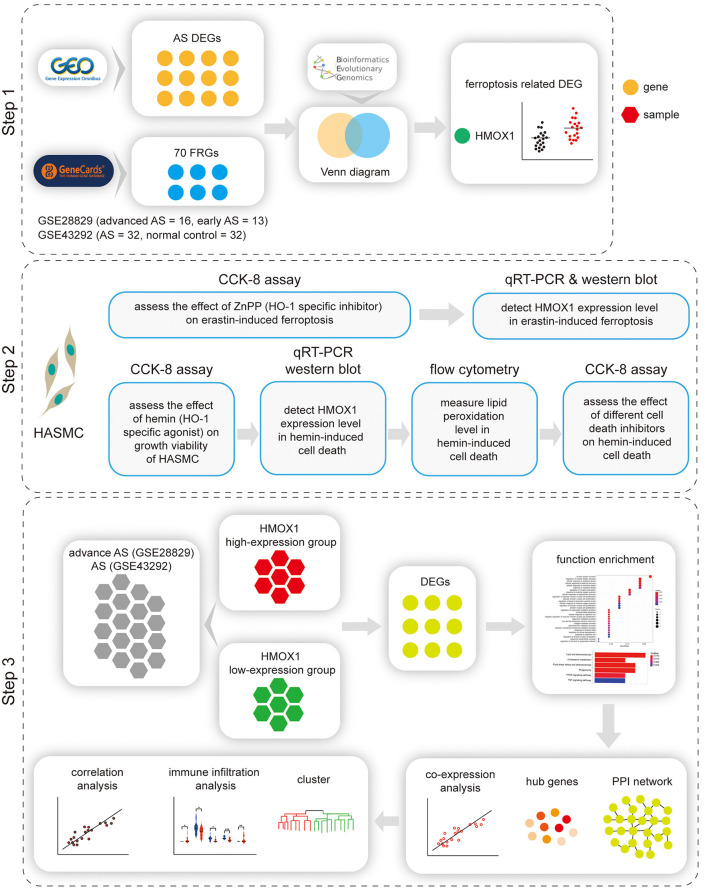
Flow chart of the study. Step 1: Identification of HMOX1 as the ferroptosis-related DEG in atherosclerosis. Step 2: Validating the pro-ferroptotic effect of HMOX1 in HASMCs. Step 3: Single-gene analysis of HMOX1. DEG, differentially expressed gene; HASMC, human aortic smooth muscle cell; AS, atherosclerosis.

A flow chart illustrating the study process is presented in Figure 1.

**Figure 2:**
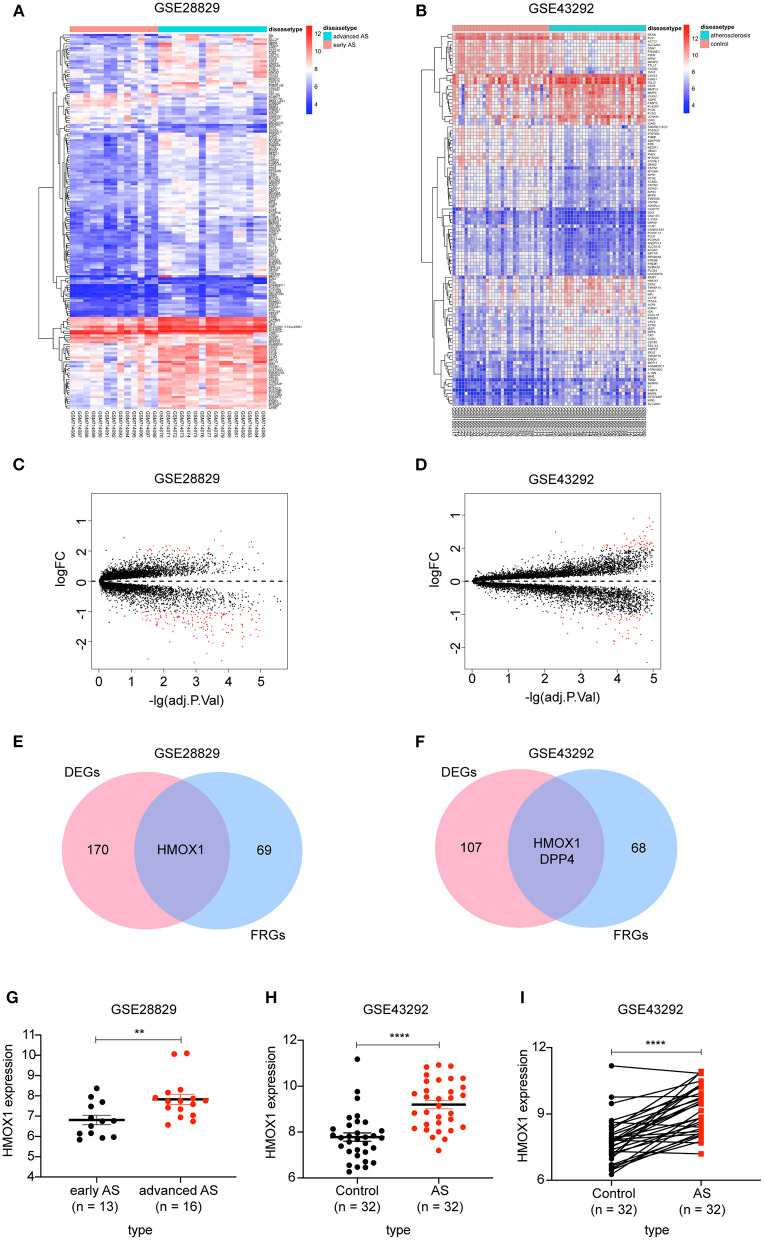
Identification of differential FRGs associated with atherosclerosis. Heatmaps showing DEGs in advanced atherosclerosis compared to early atherosclerosis in GSE28829 **(A)** and DEGs in atherosclerosis samples compared to samples of distant macroscopically intact tissue in GSE43292 **(B)**. Volcano plots of DEGs in GSE28829 **(C)** and GSE43292 **(D)**. Venn diagram showing that HMOX1 was the only FRG related to atherosclerosis resulting from the analysis of both GSE28829 **(E)** and GSE43292 **(F)** databases. The HMOX1 expression level in atherosclerotic plaques compared between paired and non-paired samples in GSE28829 **(G)** and GSE43292 **(H,I)**. (**p < 0.01, ****p < 0.0001, compared between the groups by *t*-test) (FRG, ferroptosis-related gene).

There were 171 DEGs in advanced atherosclerotic plaques compared with early plaques (Figures 2A,C) and 109 DEGs in atherosclerotic plaques compared with distant macroscopically intact tissues (Figures 2B,D). We searched GeneCards with “ferroptosis” as the keyword, and 70 genes with the association score >2 were taken into account as FRGs. Finally, there was only one FRG overlapped between DEGs in GSE28829 and 70 FRGs. And two FRGs were identified through overlapping between DEGs in GSE43329 and 70 FRGs. HMOX1, found in both of the two Venn diagrams, was recognized as an important FRG to regulate ferroptosis in atherosclerosis (Figures 2E,F).

We then assessed HMOX1 mRNA expression levels in the two GEO datasets and found significantly higher HMOX1 expression levels in advanced atherosclerotic plaques than early atherosclerotic plaques (Figure 2G), as well as higher in atherosclerosis tissues compared to normal tissues (Figures 2H,I). Through the above analysis, we identified HMOX1 as an important FRG, which might be a ferroptosis biomarker in atherosclerosis.

### Inhibitor of HO-1 Protects HASMCs From Erastin-Induced Ferroptosis

**Figure 3:**
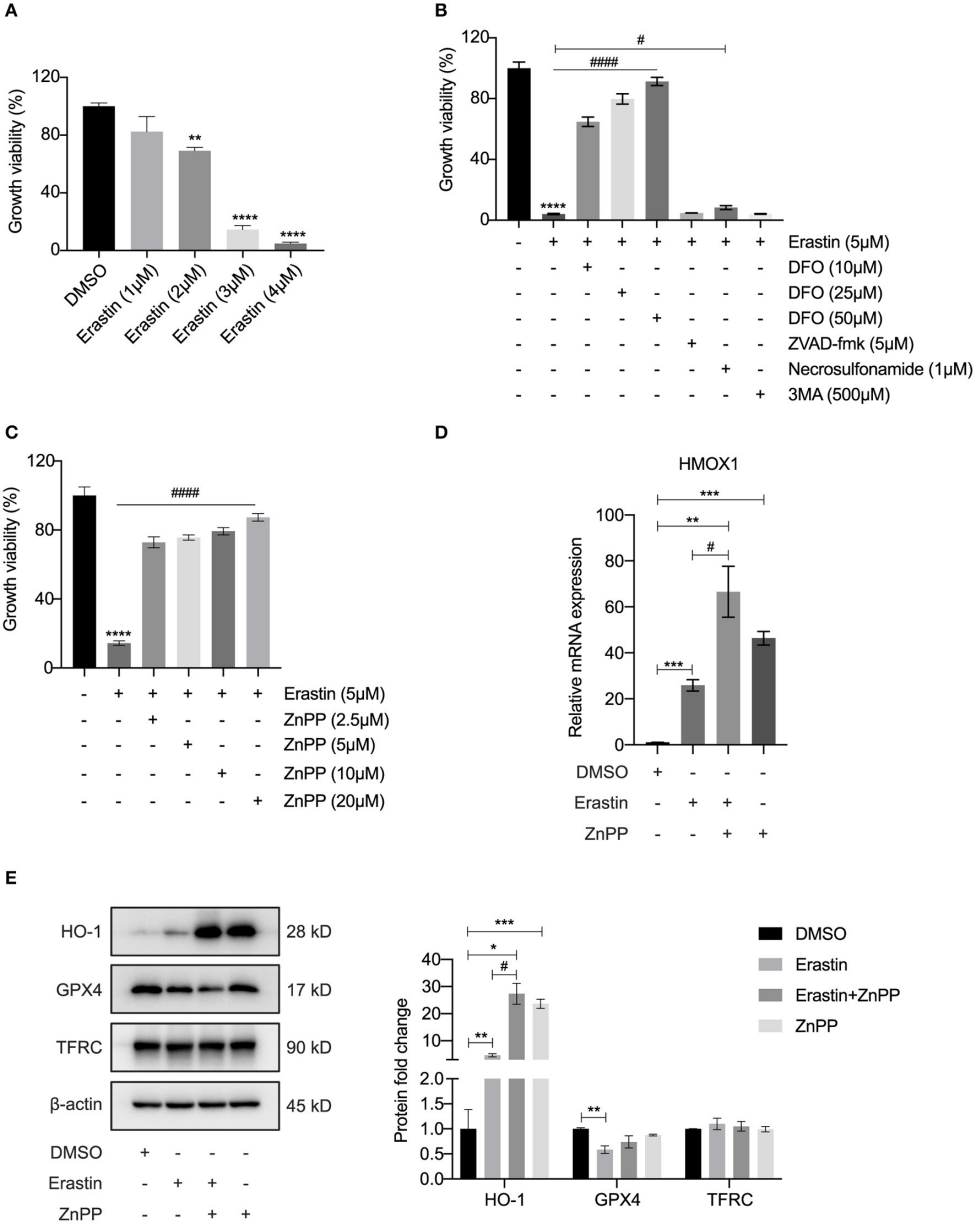
Inhibitor of HO-1 protects HASMCs from erastin-induced ferroptosis. Growth viability of HASMCs **(A)** in response to different doses of erastin for 24 h. Growth viability of HASMCs **(B)** after treatment with erastin (5 μM) in combination with different doses of DFO or different kinds of cell death inhibitors of zVAD-fmk (5 μM), necrosulfonamide (1 μM) and 3MA (500 μM) for 24 h. Growth viability of HASMCs **(C)** after treatment with erastin (5 μM) in combination with ZnPP, a classical HO-1 inhibitor. The mRNA content of HMOX1 **(D)** measured after treatment with DMSO (as control), erastin (3 μM), erastin (3 μM) combined with ZnPP (5 μM) or ZnPP (5 μM). Representative western blot images and quantitative analysis results **(E)** of HO-1, GPX4 and TFRC proteins after treatment with DMSO (as control), erastin (3 μM), erastin (3 μM) combined with ZnPP (5 μM) or ZnPP (5 μM). (The error bars represent standard error of mean from three replicates. ^#^*p* < 0.05, ^*####*^*p* < 0.0001, *p < 0.05, **p < 0.01, ***p < 0.001, ****p < 0.0001, compared between the groups by *t*-test).

As a recognized FRG, the function of HMOX1 in ferroptosis of vascular smooth muscle cells has not been reported and still requires experimental validation. In order to investigate the relationship between HMOX1 and ferroptosis, we conducted a ferroptotic model by using the classical inducer, erastin. As shown in Figure 3A, the growth viability of HASMCs declined in a dose-dependent manner by erastin treatment. To verify that the decrease of cell viability was due to iron-dependent cell death, we next observed the cell viability after treatment with erastin in the presence of an iron chelator, deferoxamine (DFO). DFO was able to rescue erastin-induced cell death in a dose-dependent manner (Figure 3B). Other cell death inhibitors had little or no effect on reversing the decrease of cell viability in HASMCs initiated by erastin (Figure 3B). Firstly, zVAD-fmk, a well-established general pan-caspase inhibitor to block apoptosis (26), failed to defend against erastin-induced cell death. Secondly, the MLKL inhibitor necrosulfonamide, which was employed to block necroptosis (27), could only slightly abolish erastin-induced cell death. Finally, 3MA, a widely used inhibitor of autophagy via its inhibitory effect on class III PI3K (28), did not affect cell death triggered by erastin. HMOX1 gene encodes heme oxygenase-1 (HO-1), which is the rate-limiting enzyme that catalyzes oxidative catabolism of heme to free iron, biliverdin, and carbon monoxide (CO) (29). The cell death triggered by erastin was almost entirely reversed by ZnPP (Figure 3C), a specific inhibitor commonly used to block the enzyme activity of HO-1 (30). The mRNA level of HMOX1 increased by almost 30-fold in the presence of erastin (Figure 3D). As is shown in Figure 3E, Erastin could trigger overexpression of HO-1 and suppress GPX4, a key enzyme to reduce lipid hydroperoxides within biological membranes (31). It has been reported that ZnPP strongly inhibits HO-1 enzyme activity, leading to a compensatory transcriptional up-regulation of HMOX1 (32). We also observed that the HMOX1 mRNA content (Figure 3D) and the protein level of HO-1 (Figure 3E) were significantly up-regulated after treatment with ZnPP. ZnPP was unable to reverse reduced GPX4 expression caused by erastin treatment (Figure 3E), which means inhibition of HO-1 protects against ferroptosis independently of GPX4. Although a recent study declared that TFRC was a protein marker of ferroptosis (33), the TFRC protein levels appeared to remain stable after different treatments in our experiment (Figure 3E). These results above demonstrated that HMOX1 is indispensable for erastin-induced ferroptosis in HASMCs and might be a pro-ferroptotic gene.

### HMOX1 Overexpression Triggered by Hemin Leads to Ferroptosis of HASMCs

**Figure 4:**
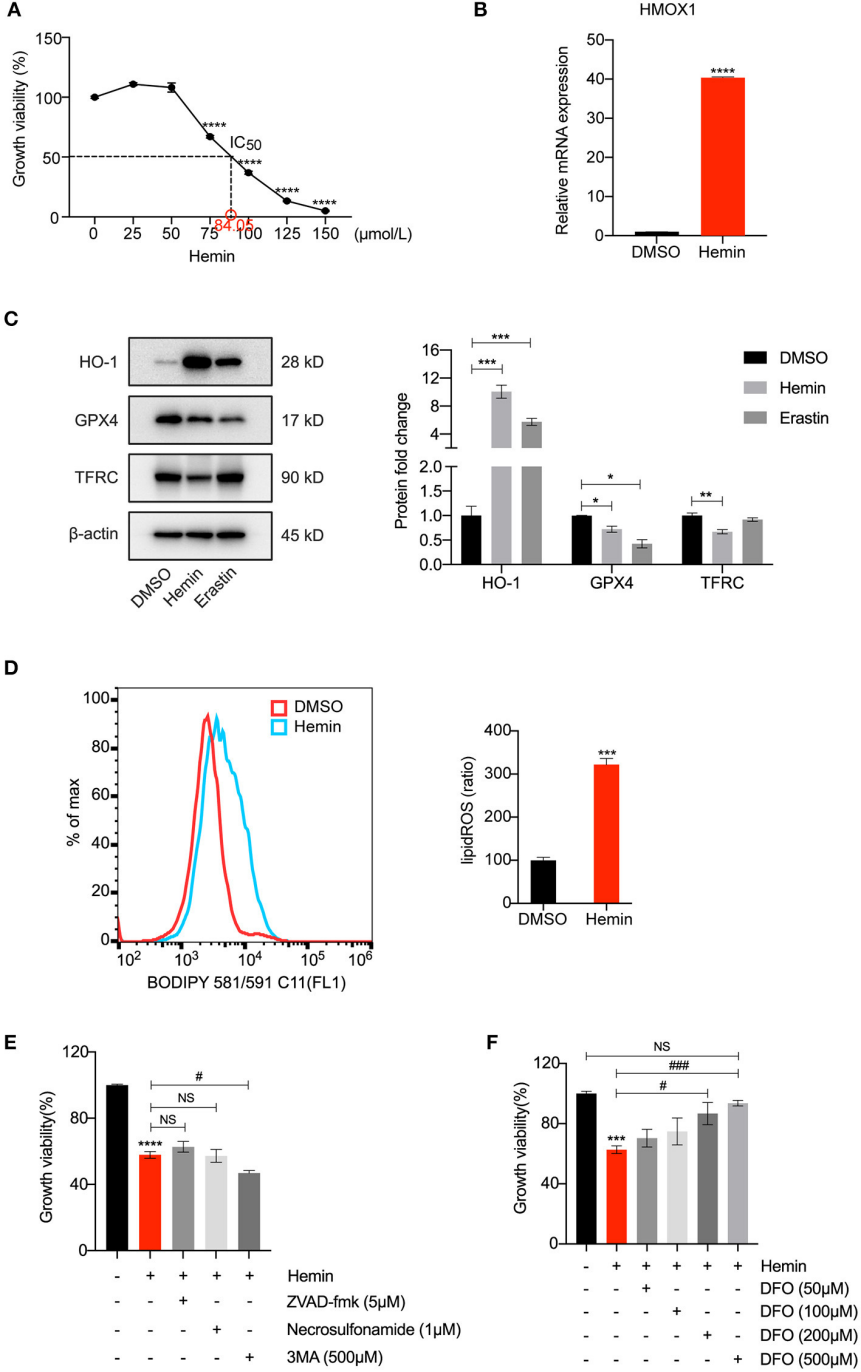
HMOX1 overexpression triggered by hemin leads to ferroptosis of HASMCs. Growth viability of HASMCs **(A)** in response to different doses of hemin for 24 h. The mRNA content of HMOX1 **(B)** detected by qRT-PCR after treatment with hemin (85 μM). Representative western blot images and quantitative analysis results **(C)** of HO-1, GPX4 and TFRC proteins after treatment with DMSO (as negative control), hemin (85 μM), and erastin (3 μM, as positive control). Lipid peroxidation production levels **(D)** detected by BODIPY 581/591 C11 stain (the ratio of lipid ROS was normalized to the blank group) after treatment with hemin (85 μM). Growth viability of HASMCs **(E)** after treatment with hemin (85 μM) in combination with different kinds of cell death inhibitors of zVAD-fmk (5 μM), necrosulfonamide (1 μM) and 3MA (500 μM) for 24 h. Growth viability of HASMCs **(F)** after treatment with hemin (85 μM) in combination with different doses of DFO. (The error bars represent standard error of mean from three replicates. NS means no significance, ^#^*p* < 0.05, ^*###*^*p* < 0.001, *p < 0.05, **p < 0.01, ***p < 0.001, ****p < 0.0001, compared between the groups by *t*-test).

To further test our conjecture, we observed the characteristic indicators related to ferroptosis in HASMCs treated with hemin, including GPX4 levels and lipid peroxidation. As shown in Figure 4A, treatment with hemin led to dose-dependent cell death in HASMCs. The mRNA level of HMOX1 increased after hemin administration (Figure 4B). The protein level of HO-1 was dramatically up-regulated compared to the control, and the increase was more significant than the erastin-treated group. Similar to erastin, hemin could also decrease the protein level of GPX4 (Figure 4C). However, unlike erastin treatment, the expression of TFRC was down-regulated when treated with hemin (Figure 4C), which might result from the increasing content of cellular free iron (34). A higher level of lipid peroxidation was observed in the hemin-treated group (Figure 4D). In addition, some inhibitors were applied to exclude other forms of cell death. Co-treatments with zVAD-fmk, necrosulfonamide and 3MA were unable to restore growth viability in HASMCs (Figure 4E). In contrast, DFO was able to reverse cell death induced by hemin in a dose-dependent manner (Figure 4F). It indicated that HMOX1 overexpression triggered by hemin caused accumulation of free iron by which hydroxyl free radicals generated from Fenton reaction (35), thus inducing the ferroptosis of HASMCs.

### The Possible Function of HMOX1 Is Explored by Conducting GO and KEGG Analysis

**Figure 5:**
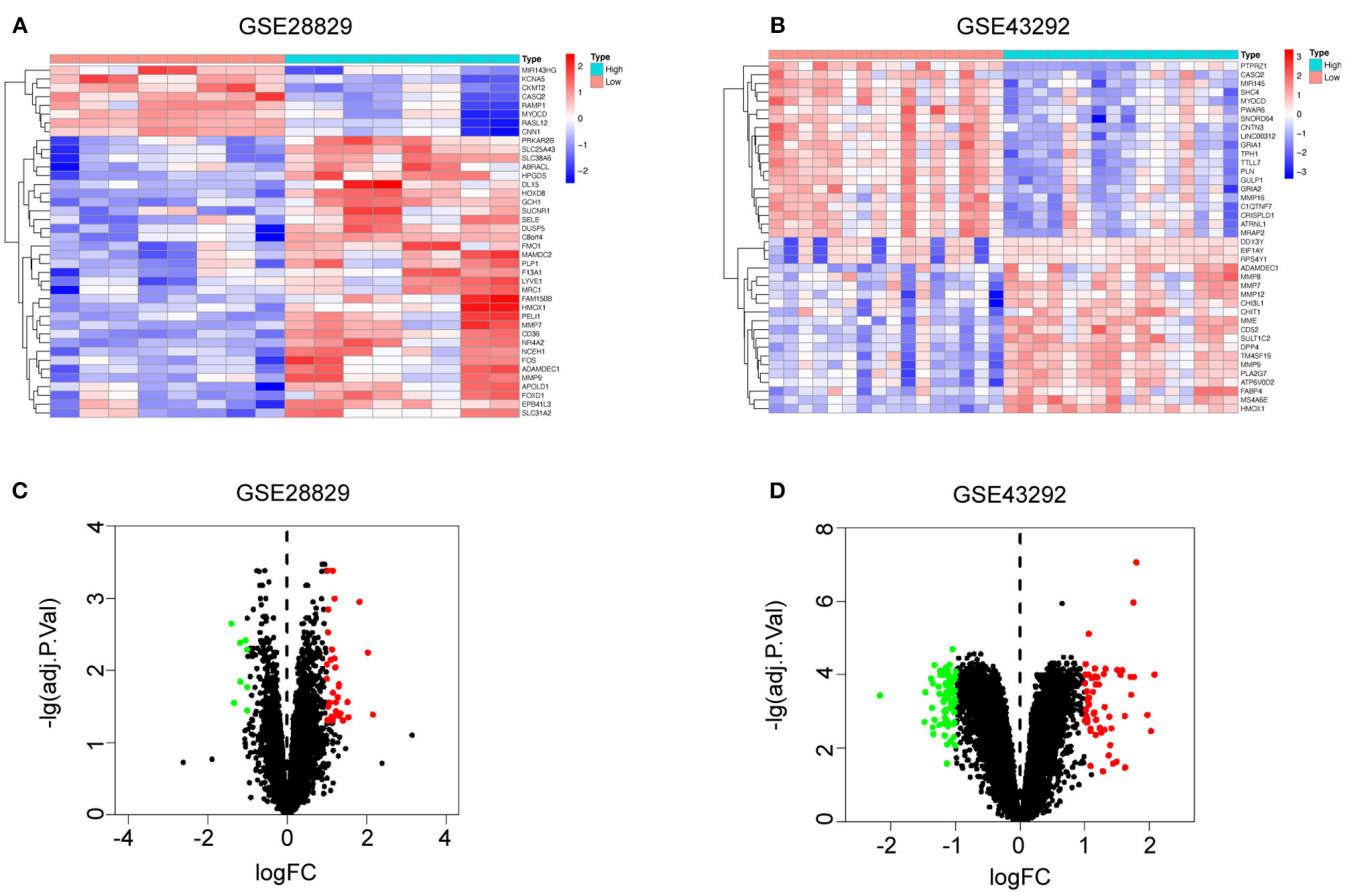
Single-gene analysis of HMOX1 in atherosclerotic plaques. Heatmaps showing DEGs in HMOX1 high-expression group compared to HMOX1 low-expression group in GSE28829 **(A)** and GSE43292 **(B)**. Volcano plots of DEGs in HMOX1 high-expression group compared to HMOX1 low-expression group in GSE28829 **(C)** and GSE43292 **(D)**.

**Figure 6:**
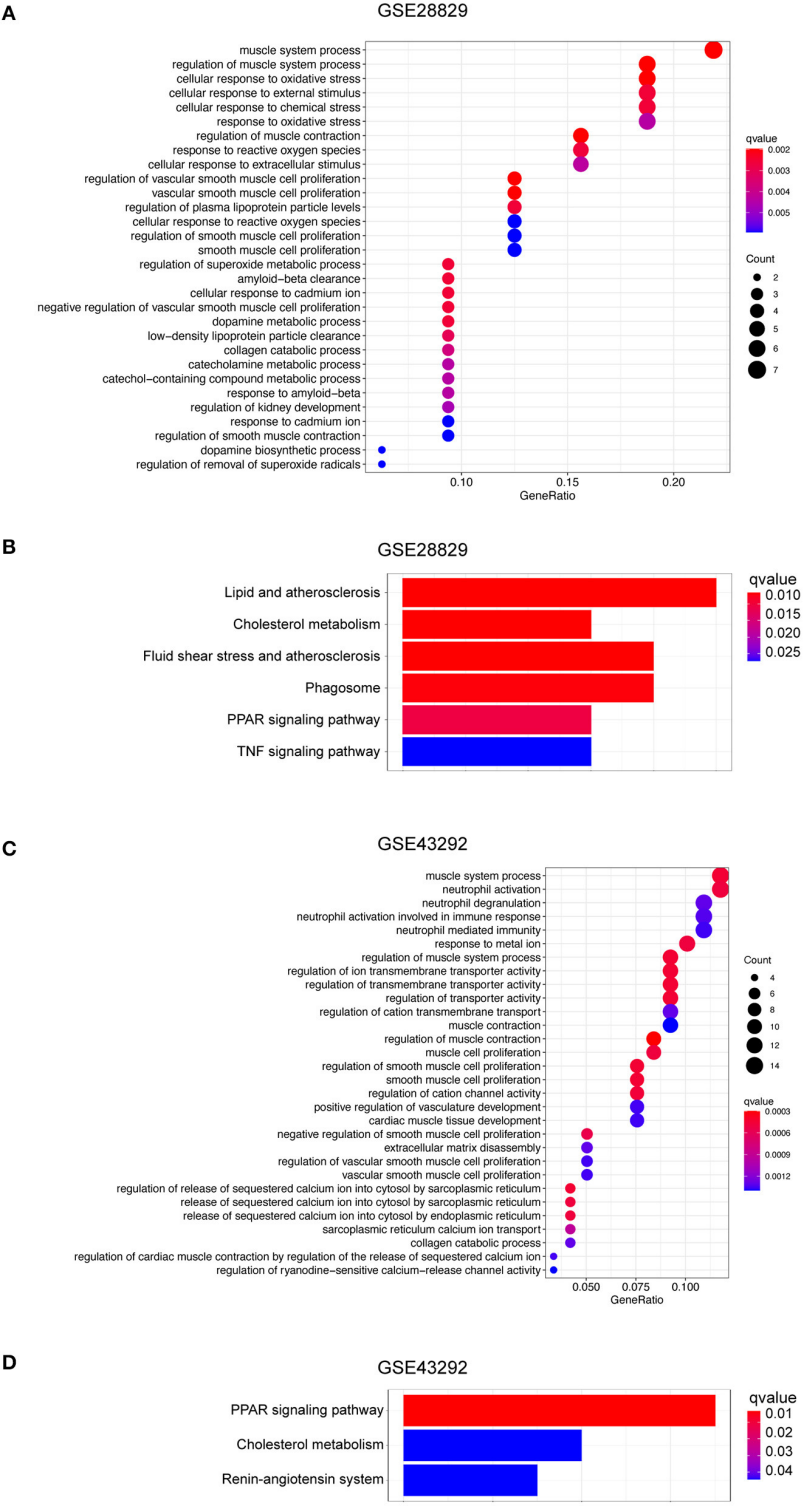
Function and pathway enrichment analysis of DEGs in HMOX1 high-expression group. The GO **(A)** and KEGG **(B)** enrichment analyses of DEGs in GSE28829. The GO **(C)** and KEGG **(D)** enrichment analyses of DEGs in GSE43292.

Based on the evidence mentioned above, we went through a single-gene analysis of HMOX1. We divided samples of advanced atherosclerotic plaques in GSE28829 and atherosclerotic plaques in GSE43292 into HMOX1 high-expression group and HMOX1 low-expression group. Heatmaps and volcano plots showed 40 DEGs with the maximum absolute value of log2FoldChange (Figures 5A–D). GO and KEGG analysis of DEGs were performed to predict HMOX1 related cell metabolism and molecular interactions among genes. GO analysis contains three domains: biological process (BP), cellular component (CC) and molecular function (MF) (36). The top 30 enriched GO terms of GSE28829 were listed in Figure 6A. The most significant GO terms were about muscle system process, cellular response to oxidative, external stimulus, chemical stress and reactive oxygen species (ROS), etc. In addition, we also found that DEGs were significantly enriched in the pathway of the regulation of vascular smooth muscle cell proliferation, which indicated that HMOX1 expression was essential to biological changes of VSMCs. The KEGG result of GSE28829 listed in Figure 6B showed that DEGs were primarily enriched in lipid and atherosclerosis, cholesterol metabolism, fluid shear stress and atherosclerosis, phagosome, PPAR signaling pathway and TNF signaling pathway, respectively. Likewise, GO analysis of GSE43292 also found that many DEGs were enriched in muscle system process and vascular smooth muscle cell proliferation regulation. Some other GO terms, such as neutrophil activation in immune response and response to metal ion, were significant (Figure 6C). PPAR signaling pathway, cholesterol metabolism and renin-angiotensin system which have a tight connection with the development of atherosclerosis, were found significant and listed in Figure 6D.

### Positive Correlation Between HMOX1 and MMPs Is Found in Atherosclerosis

**Figure 7:**
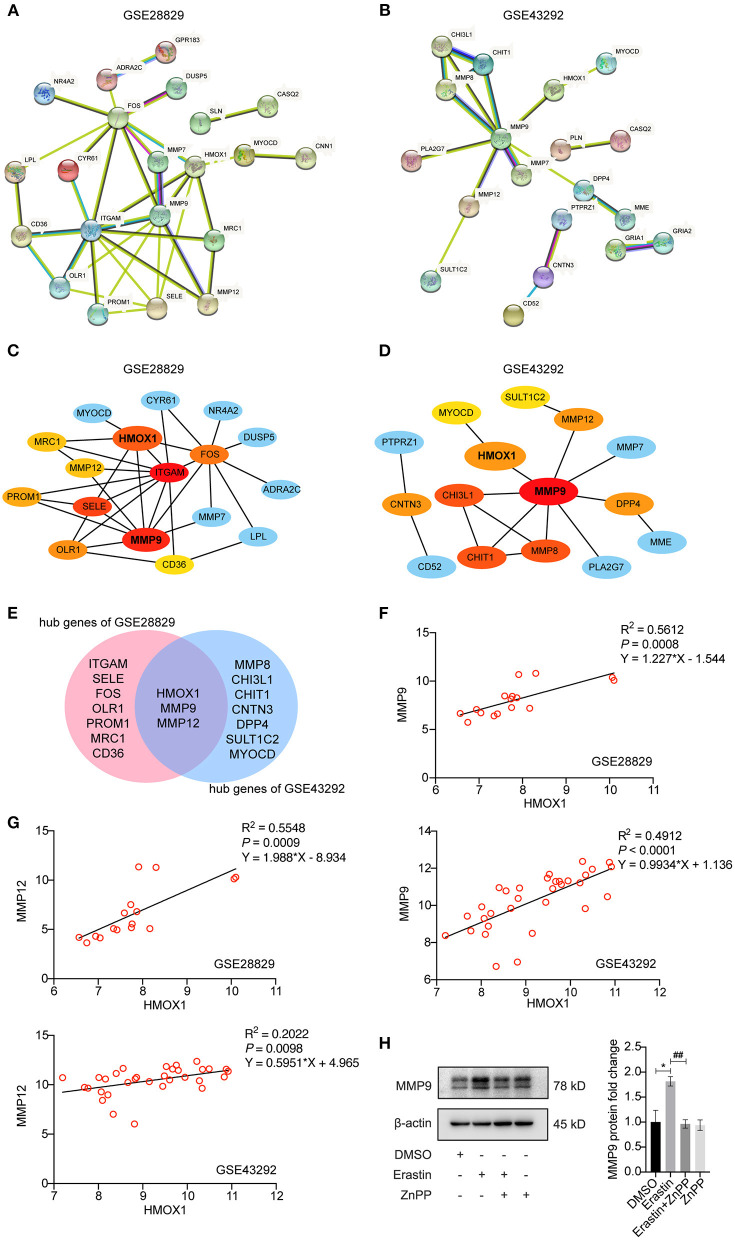
Analysis of protein-protein interaction (PPI) of DEGs in HMOX1 high- and low-expression groups. The PPI networks of DEGs identified between HMOX1 high-expression group and HMOX1 low-expression group in GSE28829 **(A)** and GSE43292 **(B)**. The hub genes identified from the PPI networks **(C,D)**. Venn diagram showing that HMOX1, MMP9 and MMP12 were hub genes **(E)**. Correlation between HMOX1 and MMP9 as well as MMP12 **(F,G)**. Representative western blot images and quantitative analysis results **(H)** of MMP9 after treatment with DMSO (as control), erastin (3 μM), erastin (3 μM) combined with ZnPP (5 μM) or ZnPP (5 μM). (The error bars represent standard error of mean from three replicates. ^*##*^*p* < 0.01, *p < 0.05, compared between the groups by *t*-test).

STRING-based network prediction analysis of 40 DEGs was performed, considering a minimum required interaction score of 0.40 (medium confidence) and hiding all disconnected nodes. Twenty one of the 40 DEGs in GSE28829 consisted of the PPI network complex (Figure 7A). And 19 of the DEGs in GSE43292 consisted of another PPI network complex using the same screening condition (Figure 7B). We then used the CytoHubba plugin in Cytoscape to screen the core genes, and identified 10 hub genes (ITGAM, MMP9, SELE, HMOX1, FOS, OLR1, PROM1, MMP12, MRC1 and CD36) in GSE28829 via MCC algorithm analysis (Figure 7C). Hub genes of GSE43292 (MMP9, MMP8, CHI3L1, CHIT1, HMOX1, MMP12, CNTN3, DPP4, SULT1C2 and MYOCD) were also identified in the same way (Figure 7D). Both of the two hub gene sets contained HMOX1, MMP9 and MMP12 (Figure 7E). The results of correlation and logistic regression analysis showed that HMOX1 had a good positive correlation with MMP9 and might be positively correlated with MMP12 (Figures 7F,G). Since HMOX1 was identified as a pro-ferroptotic gene in atherosclerosis, it would be interesting to confirm the possible relationship between ferroptosis of VSMCs and MMPs producing. Our predictions were partially validated by experimental investigation. In the presence of erastin, the protein level of MMP9 increased significantly in HASMCs, which was blocked when inhibiting HO-1 activity using ZnPP (Figure 7H).

### HMOX1 Expression Significantly Correlates With M0 Macrophage Infiltration in Atherosclerosis

**Figure 8:**
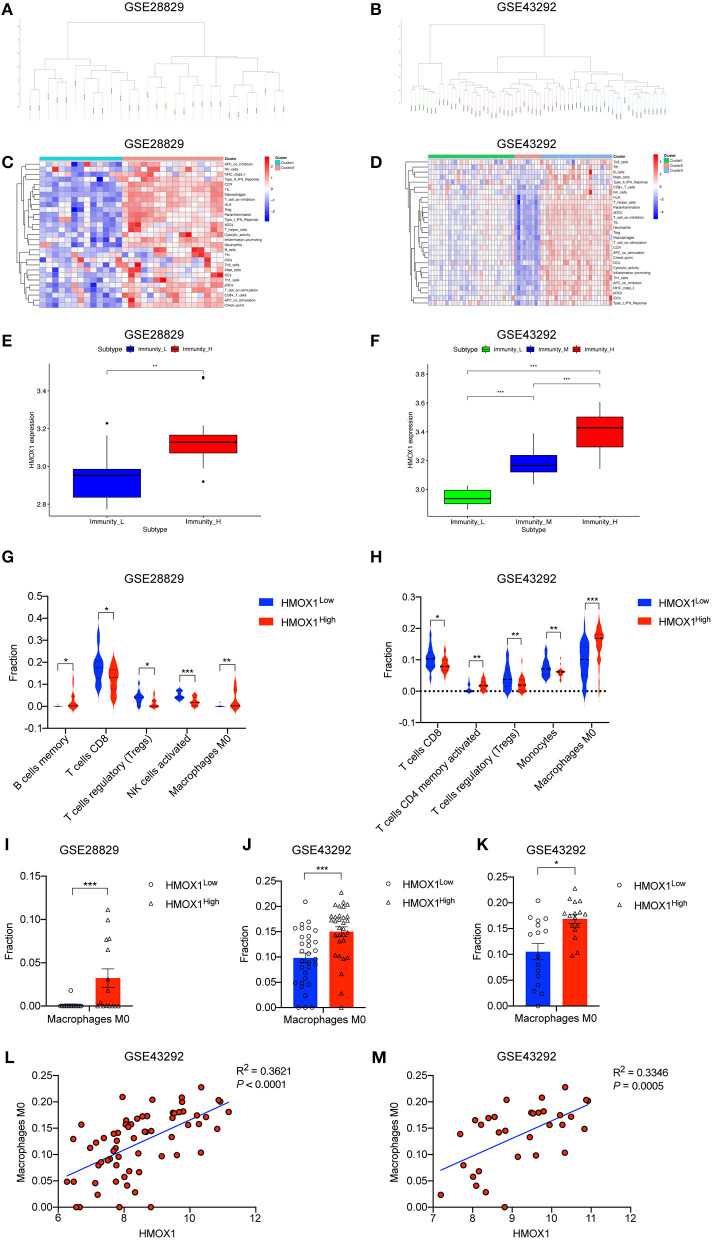
ssGSEA analysis showing high-expression of HMOX1 was related to a stronger immune response and macrophage infiltration. Samples in GSE28829 and GSE43292 clustering by immune scores **(A,B)**. Heatmaps of different immune subtypes based on ssGSEA scores for 29 immune gene sets **(C,D)**. HMOX1 expression levels among different immune subtypes **(E,F)**. Differences in the levels of immune cells (memory B cells, CD8+ T cells, regulatory T cells (Tregs), activated NK cells, M0 macrophages, activated memory CD4+ T cells, and Monocytes) among the HMOX1 high- and low-expression groups **(G,H)**. Different M0 macrophages infiltration levels among the HMOX1 high- and low-expression groups [**(I)** analysis of all atherosclerosis samples in GSE28829; **(J)** analysis of all 64 samples in GSE43292; **(K)** analysis of 32 atherosclerosis samples in GSE43292). Correlation analysis between HMOX1 expression and M0 macrophage levels among all 64 samples **(L)** and 32 atherosclerosis samples **(M)** in GSE43292. (*p < 0.05, **p < 0.01, ***p < 0.001, compared between the groups by *t*-test).

**Figure 9:**
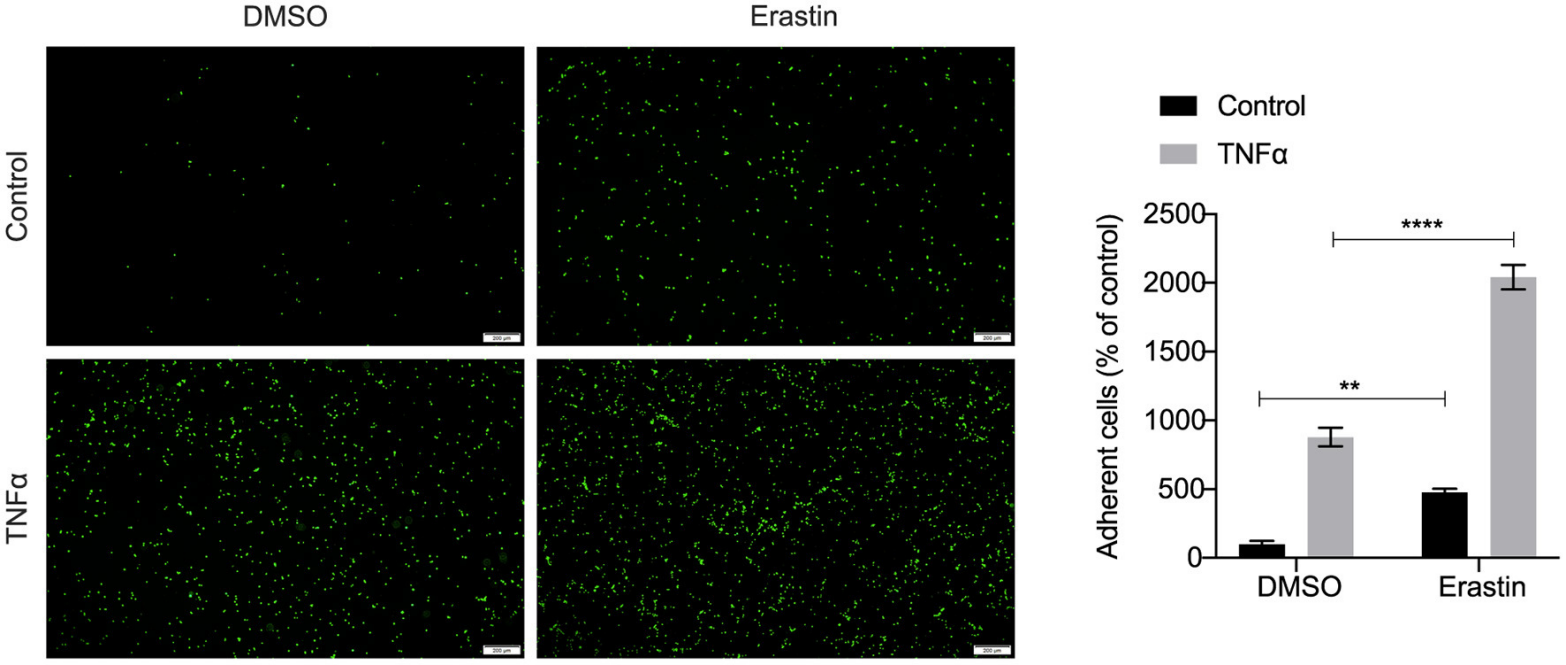
Representative pictures of the adhesion of fluorescently labeled THP-1 cells on HASMC monolayers. HASMCs were treated with DMSO (as control) or erastin (2 μM) for 24 h, then stimulated with or without TNFα (10 ng/ml) for 6 h and THP-1 cells, labeled with a fluorescent dye Calcein AM (5 μM), were added to HASMCs. After adhering, THP-1 cells bound to HASMCs were photographed by fluorescent microscopy and counted using Image J software. (The error bars represent standard error of mean from five images per well. **p < 0.01, ****p < 0.0001, compared between the groups by *t*-test).

We performed an unsupervised clustering analysis of 29 immune-associated gene sets. Based on the ssGSEA scores of the gene sets, samples of GSE28829 were divided into two groups: Immunity_H (*n* = 16, 55.17%) and Immunity_L (*n* = 13, 44.83%) (Figure 8A). And samples of GSE43292 were divided into three groups: Immunity_H (n=25, 39.06%), Immunity_M (n=30, 46.88%) and Immunity_L (*n* = 9, 14.06%) (Figure 8B). As shown in the heatmaps, samples in Immunity_H group expressed higher immune-related genes than Immunity_M and Immunity_L groups (Figures 8C,D). Evaluation of HMOX1 expression in both datasets showed that HMOX1 levels increased from the Immunity_L group to the Immunity_H group (Immunity_L < Immunity_M < Immunity_H in GSE43292) (Figures 8E,F). Then we analyzed the infiltration degrees of 22 immune-related cell types among HMOX1 high- and low-expression groups. In GSE28829, five cell types were found significantly different, including three down-regulated cells (CD8+ T cells, regulatory T cells and activated NK cells) and two up-regulated cells (memory B cells and M0 macrophages) (Figure 8G). Similarly, we identified that five types of immune-related cells showed significant differences between the two groups in GSE43292. Less infiltration of CD8+ T cells, regulatory T cells, monocytes and more infiltration of activated memory CD4+ T cells, as well as M0 macrophages, were found in the higher HMOX1 expression samples (Figure 8H). Based on these findings, M0 macrophages were selected for further analysis. Whether all samples were divided into two groups in GSE28829 and GSE43292, or only the samples derived from atherosclerotic plaques in GSE43292 were divided into two groups according to expression levels of HMOX1, higher degrees of M0 macrophage infiltrated into atherosclerotic plaques in HMOX1 high-expression groups (Figures 8I–K). This was further validated by correlation analysis (Figures 8L,M). The results showed that HMOX1 expression level was positively correlated with M0 macrophage infiltration. Since we already identified HMOX1 as an important pro-ferroptotic gene, it was warranted to investigate the relationship between ferroptosis and inflammation. Thus, the THP-1 cells adhesion assay was performed. In the presence of erastin, the adhesion of THP-1 cells to HASMCs significantly increased, although it was less effective than TNFα, which has been verified to stimulate vascular inflammatory responses through stimulating the expression of vascular cell adhesion molecule-1 (VCAM-1) in VSMCs (37). Co-administration of erastin and TNFα showed a significantly stronger effect than treatment with TNFα individually (Figure 9).

## Discussion

Since Dixon et al. introduced the ferroptosis concept in 2012 for the first time (38), the relationship between ferroptosis and various diseases has been extensively studied worldwide (39–41). Many studies have shown that ferroptosis play a crucial role in cardiovascular diseases, including atherosclerosis (11, 12), myocardial infraction (MI) (42), ischemia/reperfusion (I/R) (43, 44), heart failure (HF) (45) and so on. FRGs have been found to be associated with the progression of cardiovascular disease. For example, GPX4 was downregulated in the early and middle stages of MI, resulting in the accumulation of lipid peroxidation and ferroptosis of H9c2 cardiomyoblasts (46). Which FRGs are related to atherosclerosis remains unclear till now. Clarifying the potential FRGs and their correlation with physiological and pathological processes of atherosclerosis may provide novel biomarkers and ideas for the diagnosis, prognosis, and therapy of atherosclerosis.

We for the first time explored the ferroptosis-related DEGs in advanced atherosclerosis vs. early atherosclerosis using GSE28829, as well as in atherosclerosis vs. normal control using GSE43292. Both datasets identified HMOX1 as a key FRG which remarkably increased as atherosclerosis progressed. HMOX1 plays an essential role in doxorubicin (DOX)-induced ferroptosis in cardiomyopathy (47) and has also been considered as a pro-ferroptotic gene in some other disease models (48, 49). A recent study treated primary mouse aortic endothelial cells (MAECs) and human umbilical vein endothelial cells (HUVECs) with high glucose/high lipids (HGHL). It unveiled that HMOX1 plays a pivotal role in regulating diabetes-induced ferroptosis in endothelial injury (50). Consistently, our study confirmed that HMOX1 also displays a pro-ferroptotic effect on VSMCs. Beyond this point, we further investigated the potential relationship between ferroptosis and pathophysiological processes in atherosclerotic progression, such as immunity and inflammation.

Given the conjecture that HMOX1 can drive a ferroptotic oxidative stress and lead to cell death of VSMCs, our present study used the specific agonist and inhibitor of HO-1. On the one hand, using the inhibitor ZnPP could completely reverse erastin-induced ferroptosis. On the other hand, the agonist hemin could trigger ferroptosis accompanied by HMOX1 overexpression, which could be inhibited by the iron chelator rather than apoptosis, necroptosis, or autophagy inhibitors. This is the first study to investigate that HMOX1 is a pro-ferroptotic gene in HASMCs.

What is the key predisposing factor that induces overexpression of HMOX1 to promote ferroptosis of VSMCs in atherosclerotic plaque regions? It has been reported that excess heme can increase HO-1 expression and cellular iron to promote cardiac ferroptosis in mice with sickle cell disease (51), giving us inspiration that heme is possible to be the factor causing HMOX1 overexpression to trigger ferroptosis. Red cells infiltrating atherosclerotic lesions through the leaky neovasculature in the vasa vasorum will undergo hemolysis and release free hemoglobin. Heme is then released from hemoglobin, and non-protein bound heme is particularly deleterious as it is hydrophobic and easily able to enter cell membranes (52). Our study demonstrated that HMOX1 overexpression triggered by hemin promoted ferroptosis in HASMCs, which supported this conjecture to some extent.

Apoptosis, necrosis, and senescence of VSMCs can lead to some consequences in atherosclerotic plaques (7). Human dead VSMCs produce more extracellular matrix (ECM) degrading proteases (e.g., MMPs) (53) and secrete many pro-inflammatory cytokines to promote macrophage infiltration into the plaque (54, 55). Based on the above research, we wanted to determine which pathologic processes HMOX1 influenced by driving ferroptosis of VSMCs. Therefore, a single-gene analysis of HMOX1 was conducted. We divided advanced atherosclerosis samples of GSE28829 and all atherosclerosis samples of GSE43292 into HMOX1 high-expression group and HMOX1 low-expression group and filtered out the 40 most significant DEGs separately. GO enrichment analysis revealed that high expression of HMOX1 might be associated with cellular response to oxidative stress and other stimuli, regulation of VSMCs proliferation, immunity, and response to metal ion. KEGG pathway enrichment of DEGs demonstrated that high expression of HMOX1 might have connections with cholesterol metabolism, phagosome, PPAR signaling pathway, TNF signaling pathway, and renin-angiotensin system, most of which were activated through the development of atherosclerosis (56–59). These results suggested that ferroptosis in VSMCs might be related to pathways mentioned above.

Through PPI network analysis and hub gene screening, we noticed that in addition to HMOX1, MMP9 and MMP12 were another two hub genes involved in both of the two datasets. MMPs are a family of zinc-dependent endopeptidases that degrade components of ECM (60). They are independent predictors of atherosclerotic plaque instability. Increased levels of MMPs in advanced CAD and acute coronary syndrome (ACS) patients are associated with future risk of cardiovascular events (61). Activated MMPs can trigger endothelial damage, angiogenesis, intima-media thickness (IMT), fibrosis and calcification, which results in arterial remodeling (62). We found a positive correlation between the expression of HMOX1 and the expression of MMPs, suggesting that ferroptosis in VSMCs might promote the production of MMPs and affect the stability of plaque in the same way as other cell death mechanisms.

We then analyzed HMOX1 expression levels in samples with different degrees of immune infiltration and found higher HMOX1 expression in high immunity subgroups, which suggested the relationship between HMOX1 and inflammation. Furthermore, the infiltration of various immune cells in plaque tissue was analyzed in HMOX1 high expression groups compared to low expression groups. Analysis of GSE28829 and GSE43292 led to the same conclusion that the non-polarized (M0) macrophages displayed a higher degree of infiltration in HMOX1 high-expression tissue than HMOX1 low-expression tissue. Previously studies have reported that macrophages play a central role in the development of atherosclerosis (63). Therefore, we proposed that the ferroptosis induced by HMOX1 overexpression might result in the release of pro-inflammatory cytokines, thereby increasing the infiltration degree of macrophages. Through adhesion assay, we found that more THP-1 cells adhered to HASMCs, which were pre-treated with erastin, thereby revealing the potential relationship between ferroptosis and inflammation in atherosclerosis.

However, our study had some limitations. First, the number of samples used for analysis is small, so it would be better to confirm our findings in a larger sample size. Second, the pro-ferroptotic role of HMOX1 was not determined by utilizing HMOX1^−/−^ mice. In the future study, administration of hemin, erastin, DOX, or other ferroptosis inducers in HMOX1^−/−^ mice may not only provide *in vivo* evidence supporting the pro-ferroptotic role of HMOX1, but also help to explore the implication of ferroptosis at various stages of atherosclerosis.

In conclusion, we identified HMOX1 as an essential pro-ferroptotic gene in VSMCs, and demonstrated that high expression of HMOX1 in atherosclerosis might indicate the occurrence of ferroptosis, resulting in MMPs releasing and M0 macrophages infiltration. Our results suggested HMOX1 as a potential diagnostic biomarker for atherosclerosis, providing more evidence about the important role of ferroptosis in atherosclerosis progression.

## Data Availability Statement

The original contributions presented in the study are included in the article/Supplementary Material, further inquiries can be directed to the corresponding author.

## Author Contributions

DW, QH, YW, and JW: conceptualization. DW, QH, and YW: methodology. DW and QH: software and data curation. DW, QH, and MJ: validation. DW: formal analysis, investigation, writing—original draft preparation, and visualization. DW, QH, ZT, and JW: writing—review and editing. QH: supervision. All authors have read and agreed to the published version of the manuscript.

## Funding

This research was funded by the National Natural Science Foundation of China (Grant No. 81670409) and Zhongnan Hospital of Wuhan University Science, Technology and Innovation Seed Fund, Project znpy2019055.

## Conflict of Interest

The authors declare that the research was conducted in the absence of any commercial or financial relationships that could be construed as a potential conflict of interest.

## Publisher's Note

All claims expressed in this article are solely those of the authors and do not necessarily represent those of their affiliated organizations, or those of the publisher, the editors and the reviewers. Any product that may be evaluated in this article, or claim that may be made by its manufacturer, is not guaranteed or endorsed by the publisher.

## Supplementary Material

Supplementary Table 1 Seventy ferroptosis-related genes (FRGs) obtained from GeneCards.

Supplementary Table 2 Primers pairs for genes expression analysis.
